# Analysis of clinical and dosimetric factors associated with severe acute radiation pneumonitis in patients with locally advanced non-small cell lung cancer treated with concurrent chemotherapy and intensity-modulated radiotherapy

**DOI:** 10.1186/1748-717X-5-35

**Published:** 2010-05-12

**Authors:** Anhui Shi, Guangying Zhu, Hao Wu, Rong Yu, Fuhai Li, Bo Xu

**Affiliations:** 1 Department of Radiation Oncology, Key Laboratory of Carcinogenesis and Translational Research (Ministry of Education), Peking University School of Oncology, Beijing Cancer Hospital & Institute, Beijing 100142, China

## Abstract

**Background:**

To evaluate the association between the clinical, dosimetric factors and severe acute radiation pneumonitis (SARP) in patients with locally advanced non-small cell lung cancer (LANSCLC) treated with concurrent chemotherapy and intensity-modulated radiotherapy (IMRT).

**Methods:**

We analyzed 94 LANSCLC patients treated with concurrent chemotherapy and IMRT between May 2005 and September 2006. SARP was defined as greater than or equal 3 side effects and graded according to Common Terminology Criteria for Adverse Events (CTCAE) version 3.0.

The clinical and dosimetric factors were analyzed. Univariate and multivariate logistic regression analyses were performed to evaluate the relationship between clinical, dosimetric factors and SARP.

**Results:**

Median follow-up was 10.5 months (range 6.5-24). Of 94 patients, 11 (11.7%) developed SARP. Univariate analyses showed that the normal tissue complication probability (NTCP), mean lung dose (MLD), relative volumes of lung receiving more than a threshold dose of 5-60 Gy at increments of 5 Gy (V5-V60), chronic obstructive pulmonary disease (COPD) and Forced Expiratory Volume in the first second (FEV1) were associated with SARP (*p *< 0.05). In multivariate analysis, NTCP value (*p *= 0.001) and V10 (*p *= 0.015) were the most significant factors associated with SARP. The incidences of SARP in the group with NTCP > 4.2% and NTCP ≤4.2% were 43.5% and 1.4%, respectively (*p *< 0.01). The incidences of SARP in the group with V10 ≤50% and V10 >50% were 5.7% and 29.2%, respectively (*p *< 0.01).

**Conclusions:**

NTCP value and V10 are the useful indicators for predicting SARP in NSCLC patients treated with concurrent chemotherapy and IMRT.

## Background

Lung cancer is the leading cause of cancer-related death in the urban areas of China, accounting for approximately 600,000 deaths per year [1]. Radiotherapy plays an important role in the treatment of lung cancer, especially in patients with unresectable tumors. Recent studies have shown that concurrent chemoradiotherapy (CCRT) produced better survival rates than the sequential administration of these two modalities [2-4]. Concurrent chemoradiotherapy has become a standard method for the management of unresectable locally advanced non-small cell lung cancer (NSCLC). Unfortunately, the longer survival is achieved at the price of greater toxicity of the lung and the esophageal mucosa [3-5].

Radiation pneumonitis is one of the most common dose-limiting toxicities in lung cancer patients receiving CCRT. Severe radiation pneumonitis is life-threatening [5,6]. Many studies showed that dose and volume of radiation to lung are associated with the risk of radiation pneumonitis, such as mean lung dose (MLD) [7-11], normal tissue complication probability (NTCP) [8,12,13] value and relative volume of lung receiving more than a threshold dose (V_dose_) [7,10,14-19]. Technology such as intensity-modulated radiotherapy (IMRT) that could reduce the dose and volume of radiation to lung would potentially decrease the risk of severe radiation pneumonitis, as demonstrated in a planning study by Musherd [20] et al. Further more, Yom et al reported that IMRT was associated with a significantly reduced radiation pneumonitis rate in NSCLC patients treated with concurrent chemotherapy and so far, this is the only study that looked at concurrent chemotherapy and IMRT [21]. More clinical evidence on using IMRT in treating lung cancer is needed.

We started using IMRT to treat lung cancer in 2005, and we evaluated clinical and dosimetric factors associated with severe acute radiation pneumonitis (SARP) in 94 patients with a diagnosis of locally advanced NSCLC treated with concurrent chemotherapy and IMRT. The results are reported here.

## Methods

### Patients

Between May 2005 and September 2006, 94 consecutive locally advanced NSCLC patients were treated with concurrent chemotherapy and IMRT in the Department of Radiation Oncology at the Peking University School of Oncology, Beijing Cancer Hospital & Institute. Patients were included if they had pathologically confirmed NSCLC and clinically staged as IIIa or IIIb (AJCC 2002), treated with concurrent chemoradiotherapy. Patients were excluded if they received amifostine during concurrent chemotherapy and IMRT.

### Treatment planning

Patients were positioned in the treatment position (generally supine with arms above their heads) and immobilized by using a patient fixation device (Pelvicast Base Plate, 35781-N1, Orfit industries) to improve the setup reproducibility during CT simulation and delivery of treatment. Treatment-planning CT scan was performed using intravenous contrast if the patient was not allergic to contrast agent. CT scans with slices 5 mm thick were obtained from the mandible to the lower edge of the liver. The CT image data were directly transferred to the IMRT planning system (Varian Eclipse Treatment Planning Systems 7.0). Radiotherapy targets were defined according to the International Commission on Radiation Units and Measurements Report Nos. 50 and 62 [22,23], and the internal target volume (ITV), which was used if the required margin for target motion was visualized using fluoroscopy, was defined as a three dimensional (3D) expansion of the CTV_primary _to account for target motion, according to tumor motion. All patients' IMRT treatment plans were designed on Varian Eclipse Treatment Planning Systems to deliver the prescribed dose (1.8-2.0 Gy once per day, 60 Gy/30 fraction/6 weeks or 63 Gy/35 fractoin/7 weeks) to 95% of the planning target volume. Five to seven fields were usually used in the treatment plan. Heterogeneity correction with Eclipse-modified Batho method was applied to all dose calculations. Lung dose-volume histograms (DVH) were computed from the 3D dose distributions. Dose limitation for OAR was defined as follows: the V20 of lung less than 31%, the V55 of esophagus less than 50%, the V40 of heart less than 40 Gy, and the maximum dose administered to the spinal cord was 40 Gy. The concurrent chemotherapy consisted of two courses of Cisplatin-based chemotherapy regimen, 49 patients in all, and Paclitaxel regimen, 45 patients in all.

### Evaluation of SARP

Patients were generally evaluated by their radiation oncologist weekly during concurrent chemoradiotherapy, 3-4 weeks after completion of treatment, every 3 months in the first two years and 6 month intervals during years three to five and once a year thereafter. Chest X-ray or CT scan was performed at each follow-up after completion of chemoradiotherapy. If patients had symptoms, such as fever, cough or shortness of breath, they would be required to have an immediate examination or intervention. A diagnosis of SARP was made with consensus by at least two of three radiation oncologists and was based on clinical symptoms and radiographic infiltrate changes corresponding to the radiation portal observed during concurrent chemoradiotherapy, within the first 6 months after treatment and in the absence of any other likely cause. SARP was defined as greater than or equal to grade 3 side effects (symptomatic, interfering with activities of daily living, O_2 _indicated) and graded according to Common Terminology Criteria for Adverse Events (CTCAE) version 3.0. [24].

### Dosimetric parameters/NTCP models

The total normal lung volume was defined as the total lung volume minus the primary GTV and volume of the trachea and main bronchi. From each lung DVH, the following dosimetric factors were extracted: V_dose_, MLD, and NTCP, as derived from the Lyman and Kutcher models. The V_dose _was defined as the percentage of total normal lung volume receiving more than a threshold dose D of radiation (V_d_), where values of D considered were 5-60 Gy in increments of 5 Gy. The MLD was calculated as the average dose to total normal lung volume [25]. For the NTCP calculations, the Lyman empiric model was used with the following parameters [9]: TD50 = 30.5 Gy, m = 0.3, and n = 1.

### Statistical analysis

We evaluated clinical and dosimetric factors associated with SARP in patients after concurrent chemoradiotherapy. The following clinical parameters were considered: gender, age, smoking and diabetes history, history of COPD, induction chemotherapy, concurrent chemotherapy regimens, performance status and forced expiratory volume in 1 second (FEV1). Dosimetric factors including MLD, V5-V60 and NTCP values were analysed. DVH data and NTCP values were collected for both lungs considered as a parallel organ, Pearson Chi-Square test was performed to compare clinical parameters between patients who developed SARP and those who did not. Univariate and multivariate logistic regression analyses were performed to evaluate data for association between clinical and dosimetric factors and SARP. All statistical tests were 2-sided and p ≤ 0.05 was considered statistically significant.

## Results

All patients were followed up more than 6 months. The median follow-up for all patients was 10.5 months (range, 6.5-24.0 months). Of the 94 patients, 11 (11.7%) develop SARP; 6 (6.4%), grade 3; 2(2.1%), grade 4; and 3 (3.2%) grade 5. The SARP occurred between 4 week and 12 week (median, 8 week) from commencement of radiation treatment. There was no significant difference in the distribution of clinical parameters between the two groups of patients who developed SARP and those who did not. However, COPD and FEV1 were significant associated with SARP (*p *< 0.05) (Table 1).

**Table 1:** Distribution of the clinical, treatment factors and their association with SARP for 94 patients

Characteristic	No. of patients	No. of RP(grade ≥ 3)	p value*
Gender			
Male	73	10	0.461
Female	21	1	
Age			
>60	53	8	0.401
≤60	41	3	
smoking history			
Yes	47	6	0.748
No	47	5	
diabetes history			
Yes	13	2	0.656
No	81	9	
chronic obstructive pulmonary disease			
Yes	11	4	0.027
No	83	7	
induction chemotherapy			
Yes	73	10	0.461
No	21	1	
concurrent chemotherapy			
NVB/DDP	28	3	0.643
TXT/DDP	21	2	
PTX	45	6	
Karnofsky performance status			
≥70	80	7	0.093
<70	14	4	
Fev1(L) **			
≥2.02	71	5	
<2.02	23	6	0.036

Abbreviation: * Comparison of clinical factors between patients who developed severe acute radiation pneumonitis and those who did not. ** forced expiratory volume in 1 second. SARP = severe acute radiation pneumonitis

In univariate analysis, NTCP, MLD and V5-V60 were associated with SARP (*p *< 0.05), and were summarized in Table 2. In multivariate analysis, NTCP (*p *= 0.001) and V10 (*p *= 0.015) were the most significant factors associated with SARP (Table 3). Table 4 shows the association between the dosimetric Parameters (NTCP/V10) and the incidence of SARP for 94 patients; the incidences of SARP in the group with NTCP > 4.2% and NTCP ≤ 4.2% were 43.5% and 1.4%, respectively (*p *< 0.01); the incidences of SARP in the group with V10 ≤50% and V10 >50% were 5.7% and 29.2%, respectively (*p *< 0.01).

**Table 2:** Univariate analysis of the dosimetric parameters(MLD, NTCP, V5-V60) for predicting development of SARP for 94 patients

Variable	Median(range)	No RP(n = 83)	RP(n = 11)	pvalue*
MLD	11.59(6.53-18.11) Gy	= 11.26, SD = 2.81	= 14.91, SD = 2.91	0.001
NTCP	2.33% (0.51-9.68%)	= 2.51, SD = 1.73	= 5.94, SD = 2.40	0.001
V5	58.73% (32.89-97.65%)	= 58.43, SD = 16.57	= 69.23, SD = 12.47	0.006
V10	42.16%(25.28-83.34%)	= 41.13, SD = 12.69	= 52.42, SD = 11.05	0.001
V15	29.53%(16.46-58.51%)	= 28.94, SD = 9.12	= 38.30, SD = 7.65	0.005
V20	18.15% (9.46-31.08%)	= 18.59, SD = 6.03	= 28.02, SD = 7.09	0.002
V25	12.96% (5.90-26.26%)	= 12.88, SD = 4.42	= 20.91, SD = 6.98	0.001
V30	10.00% (4.68-21.43%)	= 9.72, SD = 3.45	= 15.69, SD = 6.00	0.008
V35	9.92% (3.89-18.65%)	= 7.57, SD = 2.86	= 11.95, SD = 4.64	0.011
V40	8.20% (3.29-13.61%)	= 5.91, SD = 2.48	= 9.26, SD = 3.75	0.015
V45	7.42% (2.73-10.99%)	= 4.41, SD = 2.25	= 6.54, SD = 3.47	0.007
V50	7.07%(1.94-8.82%)	= 3.15, SD = 1.94	= 4.89, SD = 2.15	0.007
V55	6.75% (1.33-6.32%)	= 2.03, SD = 1.65	= 3.19, SD = 1.75	0.033
V60	5.76% (0.80-4.20%)	= 1.23, SD = 1.09	= 1.66, SD = 1.15	0.039

*Abbreviation: *MLD = mean lung dose; NTCP = normal tissue complication probability; SARP = severe acute radiation pneumonitis; * Comparison of dosimetric factors between patients who developed severe acute RP and those who did not

**Table 3:** Multivariate analysis of the dosimetric and clinical factors associated with SARP for 94 patients

Varibale	OR	95%CI	*p *value*
NTCP	10.411	1.835-56.024	0.008
MLD	3.199	0.196-52.380	0.415
V5	4.024	0.765-21.163	0.100
V10	9.023	1.910-42.625	0.005
V15	4.024	0.765-21.163	0.100
V20	2.801	0.834-9.403	0.096
V25	3.423	0.713-16.421	0.124
V30	2.613	0.759-8.995	0.128
V35	2.313	0.751-7.122	0.144
V40	2.485	0.292-21.152	0.405
V45	1.219	0.373-14.146	0.475
V50	1.613	0.229-22.824	0.318
V55	1.446	0.215-20.061	0.594
V60	1.139	0.070-18.612	0.527
COPD	0.154	0.008-3.159	0.225
FEV1	0.119	0.010-1.346	0.085

*Abbreviation: *MLD = mean lung dose; NTCP = normal tissue complication probability; SARP = severe acute radiation pneumonitis; * Univariate logistic regression analysis; COPD = chronic obstructive pulmonary disease; FEV1 = forced expiratory volume in 1 second; OR = the value of odds ratio; 95%CI = confidence interval

**Table 4:** Observed rates of SARP as a function of dosimetric parameters (NTCP/V10)

Varibale	Median(Range)	Group	No. of patients	No. of RP	*p *value*
NTCP	2.33%	≤4.20%	71	1(1.4%)	0.001
	(0.51-9.68%)	>4.20%	23	10(43.5%)	
V10	42.16%	≤50%	70	4(5.7%)	0.005
	(9.91-83.34%)	>50%	24	7(29.2%)	

*Abbreviation*: NTCP = normal tissue complication probability; SARP = severe acute radiation pneumonitis; * Multivariate logistic regression analysis.

## Discussion

To the best of our knowledge, this is the first study to evaluate clinical, dosimetric factors to predicate the risk for developing SARP in locally advanced NSCLC patients during or after concurrent chemotherapy and IMRT in Chinese population. The univariate and multivariate logistic regression analysis results suggest that NTCP and V10 were the most significant factors associated with SARP (*p *< 0.05).

Radiation pneumonitis takes place usually within 1-6 months after completion of radiation therapy [11], but it can occur as late as 14 months after radiation in few patients [19]. The clinical symptoms of radiation pneumonitis can lead to a poor quality of life for lung cancer patients. Severe radiation pneumonitis after CCRT can be life-threatening if patients are not responsive to treatment. The reported incidences of radiation pneumonitis were inconsistent because of inconsistencies in the criteria used to define radiation pneumonitis, heterogeneity in patient populations, and differences in treatment regimens and radiotherapy techniques [7,11,15,19,26-28]. The incidence of SARP is 11.7% (11/94) in our study, which was similar to that reported by Yom [21] with IMRT, less than the other results with conventional or conformal radiotherapy [15,19]. Perhaps this is because we applied IMRT techniques, which had high conformity and spared more normal lung from irradiation, and therefore may have induced a low rate of severe radiation pneumonitis. The diagnosis of radiation pneumonitis is established by a history of radiotherapy, radiographic evidence (ground-glass opacity, or consolidation changes within the radiation field), and clinical symptoms (dry or productive cough, fever, chest pain, and shortness of breath). The treatment for radiation pneumonitis largely includes oral or intravenous steroids, oxygen, antibiotics and sometimes, assisted ventilation.

At present, there are no generally accepted means to predict the individual patient's risk of developing radiation pneumonitis morbidity accurately even though many clinical and dosimetric assessment of radiation pneumonitis have been studied extensively [7-19]. However, these studies lacked IMRT dosimetry data. In our study, the patient population is quite homogeneous compared with most published studies: 100% of the patients had Stage III NSCLC, 100% of the patients are Han people, and 100% received concurrent chemotherapy and IMRT. The homogeneity of demographic data in the study allowed us to focus on radiation dosimetric factors.

There are many reported studies [7,26,27,29-31] in which the risks of radiation pneumonitis were found to be associated with a variety of clinical parameters (see Additional file 1). Sex, age, smoking history, pre-existing pulmonary disease, performance score and pulmonary function before radiotherapy have been reported to affect the risk for radiation pneumonitis [7,26,29-31]. It also has been reported [27,31] that chemotherapy, particularly when combined with thoracic radiation therapy, was associated with an increased risk for radiation pneumonitis. However, (I) in our study, we only found that COPD and FEV1 were significantly associated with SARP (*p *< 0.05), suggesting that the pulmonary function before radiotherapy and base-line pulmonary disease is critical for patients' well being after chemoradiotherapy. Our findings are consistent with that of Robnett TJ [26] and Rancati T [31]. The incidences of SARP in the group with FEV1 > 2.02L and FEV1 < 2.02L were 7.04% and 26.09%, respectively (*p *= 0.036). In addition, univariate analyses show that there is not significant difference statistically between the clinical parameters (sex, age, smoking and diabetes history, induction chemotherapy, concurrent chemotherapy regimens and PS) of patients with and without SARP.

Several reports [7,9,11,14,15,19] showed that some dosimetric factors are likely to influence the risk of radiation pneumonitis (see Additional file 2), such as MLD, NTCP and percentage volume of lung receiving more than a threshold dose (Vdose). Hernando [7] reported 201 lung cancer patients treated with 3D conformal radiotherapy, and the rate of radiation pneumonitis (all grades) was significantly correlated with NTCP, MLD and V30. Kwa [9] retrospectively analyzed 400 lung cancer patients and found MLD was significantly correlated with radiation pneumonitis (grades ≥ 2). Kim [11] reported a study in which 76 lung cancer patients were treated with 3D conformal radiation therapy. In that study, the rate of severe radiation pneumonitis (grades ≥ 3) was significantly correlated with percentage of lung volume receiving 20 Gy (V20) or 30 Gy (V30), with NTCP and MLD. SARP occurred in 45% and 37% of patients with MLD of more than 15 Gy and NTCP of 50% or more, respectively, whereas it occurred in 0% of patients with a MLD of 10 Gy or less and NTCP of less than 17%, respectively. In our study, we found the NTCP and MLD were significantly associated with the incidence of SARP. SARP occurred in 2.8% of patients in whom MLD was less than 14.1 Gy, whereas it occurred in 40.9% of patients in whom MLD was greater than 14.1 Gy. This is similarly to the other study [7,9,11]. In addition, the V5-V60, in increments of 5 Gy, were all found to be significantly associated with the incidence of SARP (see Additional file 2). These findings are consistent with many other published results reported by Wang et al. [19] (rV5-V65), Willner et al. [32] (V10, V20, V30, and V40) or Fay et al. [33] (V30, V40, and V50) to be significantly associated with the incidence of radiation pneumonitis.

In our study, although the univariate analyses show that NTCP, MLD, V5-V60, COPD and FEV1 were associated with SARP (p < 0.05) however in multivariate analysis, only NTCP (p = 0.001) and V10 (p = 0.015) were found to be the significant factors associated with SARP statistically; the incidences of SARP in the group with NTCP > 4.2% and NTCP ≤ 4.2% were 43.5% and 1.4%, respectively (p < 0.01). The incidences of SARP in the group with V10 ≤ 50% and V10 >50% were 5.7% and 29.2%, respectively (p < 0.01). While NTCP can predict the incidence of radiation pneumonitis as confirmed by several studies [7,11], it is inconvenient because of intricate mathematical calculations. However, in practice, V10 was easy to calculate directly from the DVH, and furthermore, the V10 and NTCP are highly correlated (r_s _= 0.930, p = 0.001). V10, rather than V20, as an indicator suggests that radiation damage to the lung during or after IMRT is correlated with volume more closely than that of conventional or conformal radiotherapy. This finding is coincident to results reported recently by Wang et al [19], Zhu [34] and Schallenkamp [35]. Wang et al reported that V5 was the only significant factor associated with treatment-related pneumonitis; the 1-year actuarial incidences of SARP in the group with V5 <42% and V5 >42% were 3% and 38%, respectively (p = 0.001). Schallenkamp suggested V10 and V13 to be the predictors of radiation pneumonitis risk. The incidences of Grade ≥ 2 pneumonitis in the patients with V10 ≤ 32% 32%-43% and V10 > 43% were 0%-9%, 10%-20% and >20%, respectively (p < 0.01). This finding is further confirmed by Yorke et al [10] and Gopal et al [36]. Yorke [10] reported that the incidence of radiation pneumonitis rose quickly when the MLD was higher than 10 Gy. Gopal et al [36] found a sharp loss in the diffusing capacity for carbon monoxide of normal lung exposed to 13 Gy or higher, and suggested that a small dose of radiation to a large volume of lung could be much worse than a large dose to a small volume in functional lung damage. So we think that the lung received a small dose of radiation as low as 10 Gy to a large volume is not safe. In contrast, Willner [32] et al. reported that the logistic regression curve for V10, V20, V30, and V40 showed an increasing steepness toward higher doses and an increase in steepness from V10 to V40 was more pronounced for the total lung; A 10% increase in V10 resulted in a 10% increase in pneumonitis rate, whereas a 10% increase in V40 resulted in a 20% increase in pneumonitis rate. So the investigators concluded that a small dose, such as 10 Gy, to a large volume of normal lung is preferable to a large dose, such as 40 Gy, to a small volume. However, we believe that the volume of normal lung receiving low-dose irradiation should be minimized to avoid SARP. We recommend to keep the value of V10 below 50%, so as to keep the incidence of SARP lower than 5.7%.

In conclusion, NTCP and V10 are useful indicators of risk for development of SARP in locally advanced NSCLC patients after concurrent chemotherapy and IMRT. Thoracic concurrent chemoradiotherapy should be planned with caution when the volume of normal lung receiving 10 Gy or more is large with IMRT.

## Competing interests

The authors declare that they have no competing interests in this study.

## Authors' contributions

GZ and AS participated in the design of the study and performed the statistical analysis and drafted the manuscript. HW, RY, FL and BX participated in acquisition of data. All authors read and approved the final manuscript.

## Supplementary Material

Additional file 1 **Clinical parameters predictive of risk of RP as reported in the literature**. The file contains a number of important clinical parameters predictive of risk of RP as reported in the literature. Click here for file

Additional file 2 **Dosimetric parameters predictive of risk of RP as reported in the literature**. The file contains a number of important dosimetric parameters predictive of risk of RP as reported in the literature. Click here for file
