# Supplementary material for: Analysis of clinical and dosimetric factors associated with severe acute radiation pneumonitis in patients with locally advanced non-small cell lung cancer treated with concurrent chemotherapy and intensity-modulated radiotherapy
Source: Radiat Oncol. 2010 May 12;5:35. doi: 10.1186/1748-717X-5-35 (PMC2883984; doi:10.1186/1748-717X-5-35)
Supplement: Additional file 1 — Clinical parameters predictive of risk of RP as reported in the literature. The file contains a number of important clinical parameters predictive of risk of RP as reported in the literature. [file 1748-717X-5-35-S1.DOC]

| Clinical parameters predictive of risk of RP as reported in the literature | | | | |
| --- | --- | --- | --- | --- |
| Author | No. of  Patients | RP Endpoint | Effect of Parameter on Risk of RP | |
|  |  |  | Increase | No Effect |
| Hernando^(7)^et al | 201 | All grades^**^ | Smoking, weight loss | Tumor site, FEV1,chemotherapy |
| Robnett^(26)^ et al | 144 | Grades≥3^*^ | Female, low Kps (ECOG≥1),low FEV1 (≤2.0 L) | Drug, therapy sequence, tumor site |
| Yamada^(27)^ et al | 60 | Grades≥2^*^ | Tumor site in lower lung,chemotherapy, therapy sequence | Pulmonary fibrosis |
| Claude ^(29)^ et al | 96 | All grades^†^ | Age(≥60) | Gender,Kps, chemotherapy |
| Quon^(30)^ et al | 608 | Grades≥3^**^ | ND | Age |
| Rancati^(31)^ et al | 84 | Grades≥2^††^ | Drug, COPD | Gender, History of surgery |
| Present study | 94 | Grades≥3^§^ | COPD,FEV1(≤2.02 L) | Gender, Age, Kps, Smoking, Drug |

*Abbreviation:* COPD= chronic obstructive pulmonary disease; Drug =chemotherapy drug; ECOG=Eastern Cooperative Oncology Group score; FEV1= forced expiratory volume in 1 second; KPS=Karnofsky performance status, ND=no data, therapy sequence=sequence of chemotherapy and radiation therapy (concurrent vs sequential); RP= radiation pneumonitis; ^†^Score based on the Lent-Soma scale defined by the Radiation Therapy Oncology Group (RTOG) and the European Organization for the Research and Treatment of Cancer (EORTC); ^*^Score based on Radiation Therapy Oncology Group acute radiation morbidity scoring criteria. ^**^Score based on adaptation of National Institutes of Health Common Toxicity Criteria, version 2.0.

^††^Score based on Southwest Oncology Group toxicity criteria. ^§^Score based on adaptation of National Institutes of Health Common Toxicity Criteria, version 3.0.
