# Supplementary material for: Analysis of clinical and dosimetric factors associated with severe acute radiation pneumonitis in patients with locally advanced non-small cell lung cancer treated with concurrent chemotherapy and intensity-modulated radiotherapy
Source: Radiat Oncol. 2010 May 12;5:35. doi: 10.1186/1748-717X-5-35 (PMC2883984; doi:10.1186/1748-717X-5-35)
Supplement: Additional file 2 — Dosimetric parameters predictive of risk of RP as reported in the literature. The file contains a number of important dosimetric parameters predictive of risk of RP as reported in the literature. [file 1748-717X-5-35-S2.DOC]

| Dosimetric parameters predictive of risk of RP as reported in the literature | | | | | | | | | | | | | | | |
| --- | --- | --- | --- | --- | --- | --- | --- | --- | --- | --- | --- | --- | --- | --- | --- |
| Author | RP  Endpoint | NTCP | | MLD | | V5 | | V10 | | V20 | | V25 | | V30 | |
|  |  | Para-  meter (%) | RP  Rate  (%) | Para-  meter (%) | RP  Rate  (%) | Para-  meter (%) | RP  Rate  (%) | Para-  meter (%) | RP  Rate  (%) | Para-  meter (%) | RP  Rate  (%) | Para-  meter (%) | RP  Rate  (%) | Para-  meter (%) | RP  Rate  (%) |
| Hernando^(7)^ et al (n=201) | All grades^**^ | <2.5 | 10 | <10 | 10 | - | - | - | - | - | - | - | - | ≤18 | 6 |
|  |  | 2.5-8.8 | 18 | 10-20 | 16 | - | - | - | - | - | - | - | - | >18 | 24 |
|  |  | 8.9-18 | 16 | 21-30 | 27 | - | - | - | - | - | - | - | - |  |  |
|  |  | 19-79 | 33 | >30 | 44 | - | - | - | - | - | - | - | - |  |  |
| Kwa^(9)^ et al (n=400) | Grades≥2^††^ | - | - | 0-8 | 5 | - | - | - | - | - | - | - | - | - | - |
|  |  | - | - | 8-16 | 11 | - | - | - | - | - | - | - | - | - | - |
|  |  | - | - | 16-24 | 18 | - | - | - | - | - | - | - | - | - | - |
|  |  | - | - | 24-36 | 43 | - | - | - | - | - | - | - | - | - | - |
| Kim^(11)^ et al (n=76) | Grades≥3^*^ | <17 | 0 | <10 | 0 | - | - | - | - | <32 | 0 | - | - | <25 | 0 |
|  |  | 17–49 | 8 | 10–14.9 | 10 | - | - | - | - | 32–41 | 8 | - | - | 25–34 | 11 |
|  |  | ≥50 | 37 | ≥15 | 45 | - | - | - | - | ≥42 | 40 | - | - | ≥35 | 39 |
| Graham^(14)^ et al (n=99) | Grades≥2^*^ | - | - | <10 | 0 | - | - | - | - | <22 | 0 | - | - | - | - |
|  |  | - | - | 11-20 | 9 | - | - | - | - | 22-31 | 7 | - | - | - | - |
|  |  | - | - | 21-30 | 24 | - | - | - | - | 31-40 | 13 | - | - | - | - |
|  |  | - | - | >30 | 25 | - | - | - | - | >40 | 36 | - | - | - | - |
| Tsujino^(15)^ et al (n=71) | Grades≥2^**^ | - | - | - | - | - | - | - | - | ≤20 | 8.7 | - | - | - | - |
|  |  | - | - | - | - | - | - | - | - | 21-25 | 18.3 | - | - | - | - |
|  |  | - | - | - | - | - | - | - | - | 26-30 | 51 | - | - | - | - |
|  |  | - | - | - | - | - | - | - | - | ≥31 | 85 | - | - | - | - |
| Wang^(19)^ et al (n=223) | Grades≥3^§^ | - | - | ≤16.5 | 13 | ≤42 | 3 | ≤33 | 5 | ≤28 | 4 | ≤27 | 3 | ≤22 | 10 |
|  |  | - | - | >16.5 | 36 | >42 | 38 | >33 | 37 | >28 | 37 | >27 | 38 | >22 | 36 |
| Present study(n=94) | Grades≥3^§^ | ≤4.2 | 1.4 | ≤14.1 | 2.8 | ≤69 | 7.1 | ≤50 | 5.7 | ≤25 | 2.9 | ≤17 | 2.9 | ≤12.5 | 5.6 |
|  |  | >4.2 | 43.5 | >14.1 | 40.9 | >69 | 25 | >50 | 29.2 | >25 | 25 | >17 | 25 | >12.5 | 30.4 |

*Abbreviation:* RP= radiation pneumonitis; NTCP=normal tissue complication probability; no data=-; ^*^Score based on Radiation Therapy Oncology Group acute radiation morbidity scoring criteria; ^**^Score based on adaptation of National Institutes of Health Common Toxicity Criteria, version 2.0.; ^††^Score based on Southwest Oncology Group toxicity criteria;^§^Score based on adaptation of National Institutes of Health Common Toxicity Criteria, version 3.0.
